# Maternal Fish Consumption, Hair Mercury, and Infant Cognition in a U.S. Cohort

**DOI:** 10.1289/ehp.8041

**Published:** 2005-05-26

**Authors:** Emily Oken, Robert O. Wright, Ken P. Kleinman, David Bellinger, Chitra J. Amarasiriwardena, Howard Hu, Janet W. Rich-Edwards, Matthew W. Gillman

**Affiliations:** 1 Department of Ambulatory Care and Prevention, Harvard Medical School and Harvard Pilgrim Health Care, Boston, Massachusetts, USA; 2 Department of Pediatrics, Boston Children’s Hospital and Harvard Medical School, Boston, Massachusetts, USA; 3 Channing Laboratory, Brigham and Women’s Hospital and Harvard Medical School, Boston, Massachusetts, USA; 4 Department of Neurology, Boston Children’s Hospital and Harvard Medical School, Boston, Massachusetts, USA; 5 Department of Environmental Health,; 6 Department of Epidemiology, and; 7 Department of Nutrition, Harvard School of Public Health, Boston, Massachusetts, USA

**Keywords:** child development, cognition, environmental health, fish, mercury, n-3 fatty acids, neurotoxins, seafood

## Abstract

Fish and other seafood may contain organic mercury but also beneficial nutrients such as n-3 polyunsaturated fatty acids. We endeavored to study whether maternal fish consumption during pregnancy harms or benefits fetal brain development. We examined associations of maternal fish intake during pregnancy and maternal hair mercury at delivery with infant cognition among 135 mother–infant pairs in Project Viva, a prospective U.S. pregnancy and child cohort study. We assessed infant cognition by the percent novelty preference on visual recognition memory (VRM) testing at 6 months of age. Mothers consumed an average of 1.2 fish servings per week during the second trimester. Mean maternal hair mercury was 0.55 ppm, with 10% of samples > 1.2 ppm. Mean VRM score was 59.8 (range, 10.9–92.5). After adjusting for participant characteristics using linear regression, higher fish intake was associated with higher infant cognition. This association strengthened after adjustment for hair mercury level: For each additional weekly fish serving, offspring VRM score was 4.0 points higher [95% confidence interval (CI), 1.3 to 6.7]. However, an increase of 1 ppm in mercury was associated with a decrement in VRM score of 7.5 (95% CI, –13.7 to –1.2) points. VRM scores were highest among infants of women who consumed > 2 weekly fish servings but had mercury levels ≤1.2 ppm. Higher fish consumption in pregnancy was associated with better infant cognition, but higher mercury levels were associated with lower cognition. Women should continue to eat fish during pregnancy but choose varieties with lower mercury contamination.

The developing brain is uniquely vulnerable to environmental insult (Rice and Barone 2000). During the last century, devastating community-level exposures to mercury-contaminated grain in Iraq and seafood in Japan established the sensitivity of the fetus to mercury’s toxic effects (Myers and Davidson 2000). More recently, two well-designed longitudinal studies have studied whether chronic low-level exposure to organic mercury from maternal seafood consumption during pregnancy is associated with adverse effects on fetal brain development, with conflicting results. Among 900 children in the Faroe Islands followed to 7 years of age, higher umbilical cord blood methylmercury was associated with lower scores on several developmental and cognitive tests (Grandjean et al. 1997). In contrast, in the Seychelles Child Development Study, no association of total maternal hair mercury with neurodevelopmental test performance was demonstrated in follow-up of > 700 children to 9 years of age (Davidson et al. 1998; Myers et al. 2003), despite the fact that mercury levels were similar to those in the Faroe Islands.

However, for women making dietary choices, the relevant question is whether eating fish and other seafood during pregnancy will impair their children’s development. Fish contain nutrients, including n-3 polyunsaturated acids, that may benefit mother and fetus (Lauritzen et al. 2001; Neuringer et al. 1994; Olsen et al. 1993). The net effect of the beneficial nutrients and harmful contaminants contained within fish has not been well studied and remains unclear (Chapman and Chan 2000). One recent study of 7,421 British children reported higher developmental scores at 15 months of age among offspring of women who consumed more fish during pregnancy, but found no association of umbilical cord tissue mercury levels with development among a subset of these participants (Daniels et al. 2004). In the present study, we examined associations of maternal fish and seafood intake and maternal hair mercury at delivery with 6-month infant cognition in a U.S. cohort.

## Materials and Methods

### Subjects and setting.

Study subjects were participants in Project Viva, a prospective cohort study of gestational diet and other exposures, pregnancy outcomes, and offspring health in eastern Massachusetts. Institutional review boards of participating institutions approved the study. All procedures were in accordance with the ethical standards for human experimentation established by the Declaration of Helsinki (World Medical Association 1997). Recruitment and retention procedures have been described previously (Gillman et al. 2004; Oken et al. 2004).

Briefly, we recruited women at their initial clinical obstetric appointment. Eligible participants were women who presented for their initial clinical visit at < 22 weeks of gestation, had a singleton pregnancy, were able to complete study forms in English, and did not plan to move out of the study area before delivery. We collected information about demographics, health history, and health habits by interview and self-administered questionnaire and obtained infant birth weight and gestation length from medical records (Oken et al. 2004). We calculated birth weight for gestational age using a U.S. national reference (Oken et al. 2003b).

At the second study visit, performed at 26–28 weeks of gestation, participants completed a semiquantitative food frequency questionnaire, which has been previously calibrated against blood levels of long-chain marine n-3 fatty acids (Fawzi et al. 2004). The questionnaire quantified average frequency of consumption of > 140 specified foods and beverages, including alcohol, during the preceding 3 months. The four questions regarding fish queried intake of “canned tuna fish (3–4 oz.)”; “shrimp, lobster, scallops, clams (1 serving)”; “dark meat fish, e.g., mackerel, salmon, sardines, bluefish, swordfish (3–5 oz.)”; and “other fish, e.g., cod, haddock, halibut (3–5 oz.).” Six response options ranged from “never/less than 1 per month” to “1 or more servings per day.” We combined responses for the four questions and generated a count of weekly total second-trimester fish servings. We used second-trimester diet as the measure of fish exposure because the timing of intake assessed by this questionnaire best overlapped with the timing of mercury exposure assessed by the maternal hair length sampled.

During the entire study period from April 1999 through February 2003, 2,128 participants delivered a live infant. We began collecting maternal hair samples in February 2002 and continued through the end of the study. During this period, 409 study participants delivered, of whom 302 were approached for collection of a hair sample during the hospitalization for delivery. The others were not approached mainly because they were hospitalized over a weekend only or because a trained research assistant was not available to meet them during their hospital stay. Thirty-two women were ineligible (hair too short or in braids), and 211 of the remaining 270 (78%) consented to provide a hair sample. Of these, 135 mother–infant pairs had complete information on both maternal second-trimester diet and 6-month infant cognitive testing and thus constitute the sample for the present analysis.

In the hospital after delivery, research assistants collected a sample of approximately 50–100 strands of hair from the mother’s occipital scalp, tied the hair at the proximal end, and stored it in a paper envelope at room temperature. We also asked about known exposure to mercury, for example, from occupational exposure. We did not collect information about dental amalgams or recent dental work.

### Hair mercury assay.

We analyzed the proximal 3-cm length of the maternal hair specimen for total mercury content. This hair length represents growth during approximately months 6–8 of pregnancy, because the hair produced in the month before sampling remains under the scalp (National Research Council 2000). All samples were handled in a class 100 clean hood. We precleaned plastic and glassware by soaking them in 10% HNO_3_ for 24 hr, and then rinsing them several times with deionized water. Hair samples were sonicated for 15 min in approximately 10 mL of 1% Triton X-100 solution in precleaned 50-mL Pyrex beakers. After sonication, samples were rinsed several times with distilled deionized water and dried at 60°C for 24 hr.

We performed mercury assays using the Direct Mercury Analyzer 80 (Milestone Inc., Monroe, CT). This automatic mercury analyzer requires no sample digestion or pretreatment. The cleaned sample of hair was weighed into a nickel boat, thermally decomposed, and amalgamated, and then the released mercury was measured by atomic absorption spectroscopy at 253.7 nm as a function of mercury concentration. Samples were analyzed by using a matrix-matched calibration curve created with different weights of certified reference material GBW 09101 (human hair; Shanghai Institute of Nuclear Research, Academia Sinica, China) containing 2.16 ppm mercury.

Quality control steps included daily calibration with verification of a high- and a low-concentration standard for each working range, a procedural blank, and certified reference material NIES CRM-13 (human hair; National Institute for Environmental Studies, Ibaraki, Japan) as the standard. Mercury recovery was 90–110%, with > 95% precision.

### Cognitive testing.

When the infants reached approximately 6 months of age, we performed cognitive testing using the visual recognition memory (VRM) paradigm. All subjects were first tested for visual acuity and had results within the normal range (Teller et al. 1986). We performed tests at the child’s home or in a research clinic. The infant was seated on the mother’s lap, with the mother’s view of the test stimuli shielded to minimize any possible influence on the infant’s performance.

Trained test administrators presented the infant with two identical photographs of an infant’s face, at a standardized distance. The habituation trials were repeated, with no maximum number of presentations, until the infant became habituated to this stimulus. In the testing phase, the infant was presented with the previously seen photo simultaneously with a novel photo of another infant’s face. Using a laptop computer, test administrators tracked the amount of time that the infant looked at each stimulus. The computer then calculated a novelty preference, the percentage of the total test time that the infant spent looking at the novel stimulus. The test administrator also recorded her confidence that the test was performed without distractions or any other concerns that might influence results. We excluded results for which the administrator did not have confidence in the test performance.

Each infant had two test trials, with the positions of the two faces alternated; the final score represents the average of the two trials. This test reflects the infant’s ability to encode a stimulus into memory, to recognize that stimulus, and to look preferentially at a novel stimulus (McCall and Carriger 1993), and is correlated with later IQ (Rose and Feldman 1995; Rose et al. 1992).

### Statistical analysis.

We assessed factors associated with VRM score using linear regression. We first performed individual bivariate analyses with each maternal and child characteristic. For multivariable analyses, we included as independent predictors of VRM score both maternal hair mercury obtained at delivery (hereafter referred to as “mercury level”), and maternal second-trimester weekly intake of combined fish and seafood (hereafter referred to as “fish intake”), as well as the covariates maternal age (continuous), race/ethnicity (white vs. nonwhite), education (college graduate vs. not), and marital status (married or cohabiting vs. not), and infant sex, gestational age at birth (continuous), birth weight for gestational age (continuous), breast-feeding duration (continuous), and age at cognitive testing (continuous).

We studied both mercury levels and fish intake as continuous predictors. In addition, we dichotomized fish intake and mercury exposure based on public health recommendations. Federal advisories have recommended that pregnant women should consume two or fewer weekly fish meals (U.S. Department of Health and Human Services 2004). A hair methylmercury level of 1.2 ppm has been recommended as the U.S. reference dose (National Research Council 2000). Most hair mercury is in the methyl form, and total rather than methylmercury in hair has been recommended as a biomarker for mercury exposure (Davidson et al. 1998). We explored associations with VRM score using these dichotomized measures.

All final models met standard assumptions for linear regression. We did not see any evidence of influential outliers. We performed all analyses using SAS (version 8.2; SAS Institute Inc., Cary NC).

## Results

Women in this cohort were predominantly white (82%), married or living with a partner (92%), and well educated (Table 1). The great majority of participants (94%) breast-fed their children, and half continued to breast-feed until at least 6 months postpartum. During pregnancy, 106 women (79%) consumed some alcohol, although only 25 (19%) continued consuming alcohol after learning they were pregnant; median first- and second-trimester alcohol consumption was 1.0 g/day (25th percentile 0.4, 75th percentile 3.0). Ten women (7%) reported smoking during pregnancy, and one reported illicit drug use. Use of these substances was not related to maternal hair mercury or to fish consumption. Six infants were born preterm (< 37 weeks), and three were small for gestational age (< 10th percentile).

Women consumed an average of 1.2 servings per week of combined tuna, dark meat, white meat, and shellfish (range, 0–5.5 servings/week) in the second trimester of pregnancy. The mean maternal hair mercury level was 0.55 ppm (micrograms per gram), with a range of 0.02–2.38. The geometric mean hair mercury level was 0.45 ppm. Fourteen participants (10%) had mercury levels > 1.2 ppm. No participants reported occupational exposure to mercury.

Total maternal fish intake was moderately associated with hair mercury content (Spearman *r* = 0.47). For each additional weekly total fish serving, mercury was 0.17 ppm higher [95% confidence interval (CI), 0.10 to 0.24]. Consumption of each group of fish was also correlated with hair mercury, with Spearman correlation coefficients with hair mercury ranging from *r* = 0.43 for canned tuna to *r* = 0.23 for white meat fish. No other maternal and infant sociodemographic characteristics, including breast-feeding, were associated with fish consumption or mercury levels (data not shown).

The mean VRM score (percent novelty preference) was 59.8 (range, 10.8–92.5). Infants were tested between 5.5 and 8.4 months of age (mean, 6.5 months). VRM score did not differ by maternal and infant characteristics (Table 1), including maternal use of cigarettes or alcohol during pregnancy. On bivariate analyses, maternal fish intake was positively associated with VRM score, and mercury was negatively associated with VRM score, although CIs did not exclude zero (Table 2). After adjusting for maternal and infant characteristics, each additional weekly fish serving was associated with a VRM score that was 2.8 (95% CI, 0.2 to 5.4) points higher (Table 2). In the similarly adjusted analysis, CIs for mercury did not exclude zero, although the estimate suggested that increasing mercury was associated with reduced VRM score.

To determine the independent effect of each on offspring cognition, we included both fish consumption and mercury level simultaneously in the linear regression model. In this multivariate model, both fish intake (direct) and mercury (inverse) were significantly and more strongly associated with infant cognition (Table 2). We further included as covariates maternal age, race/ethnicity, education and marital status, and infant sex, gestational age, birth weight for gestational age, breast-feeding duration, and age at cognitive testing. After adjustment, an increase of 1 ppm in maternal hair mercury was associated with a decrement in VRM score of 7.5 (95% CI, –13.7 to –1.2) points. For each additional weekly fish serving consumed by the mother, offspring VRM score was 4.0 (95% CI, 1.3 to 6.7) points higher on similarly adjusted analysis. Exclusion of participants with extreme values for mercury, fish intake, or VRM score did not markedly change results. Results were similar among nonsmokers only, and among nondrinkers only (data not shown).

We also examined multivariate associations with VRM score according to recommended thresholds for fish intake and mercury exposure. After adjustment for participant characteristics and mercury (continuous), the nine participants (7%) who consumed more than two weekly fish servings had infants with VRM scores that were 12.0 (95% CI, –0.1 to 24.1) points higher than those who consumed two or fewer weekly servings. Offspring of the 14 mothers (10%) with hair mercury > 1.2 ppm had VRM scores 9.3 (95% CI, –19.3 to 0.8) points lower than those with hair mercury ≤1.2 ppm, after adjustment for participant characteristics and fish intake (continuous).

We next investigated whether an interaction existed between low fish consumption and high mercury levels. Because of small numbers, we were not able to perform a multivariate analysis including both dichotomized variables. However, unadjusted VRM scores appeared highest among infants of mothers with high fish intake and low mercury levels, whereas scores appeared lowest in infants of mothers with low fish intake and high mercury (Table 3). We lacked power to demonstrate a statistically significant interaction in this unadjusted analysis.

## Discussion

These results support findings from some studies that higher mercury exposure in pregnancy is associated with lower offspring cognitive scores, even at these relatively low levels of exposure. In addition, higher maternal fish intake was associated with higher mercury levels. However, higher maternal fish consumption was associated with better infant cognition. This benefit appeared greatest among infants whose mothers consumed more fish but had lower mercury levels.

The conflicting results from the two large-scale longitudinal studies in the Faroe and Seychelles Islands have led to disagreement about whether moderate mercury exposure from frequent seafood consumption may harm offspring development. The mean maternal hair mercury levels in the Faroe Islands (4.3 ppm) (Grandjean et al. 1997) and Seychelles Islands (6.8 ppm) (Davidson et al. 1998) studies were much higher than in our cohort (0.55 ppm), which had levels similar to other pregnant (Morrissette et al. 2004; Stern et al. 2001) and nonpregnant (Centers for Disease Control and Prevention 2001) U.S. populations. Some data suggest that no threshold exists for adverse neuropsychologic effects from methylmercury exposure (Rice 2004). A recent study in the United Kingdom did not show any adverse association of low levels of umbilical cord tissue mercury (median, 0.01 ppm) with child development (Daniels et al. 2004). However, in the U.K. study, development at 15 months of age was assessed by parental self-report, which is not likely to be as sensitive to the adverse effects of mercury.

Nevertheless, because high-dose organic mercury is known to harm the developing fetus, and because fish contain organic mercury, advisory bodies in the United States (National Research Council 2000; U.S. Department of Health and Human Services 2004), Canada (Health Canada 2002), and the United Kingdom (Committee on Toxicity 2004) have recommended that pregnant women limit their fish consumption. However, fish also contain nutrients such as iron, vitamin E, selenium, and long-chain n-3 polyunsaturated fatty acids that may benefit brain development (Clarkson and Strain 2003; National Research Council 2000; Neuringer et al. 1994). Little information has been available about the balance of risk and benefit for fish consumption.

It may seem contradictory that, on the one hand, fish intake raises mercury levels and higher mercury levels lead to worse cognition but, on the other hand, higher fish consumption is associated with better cognition. The most likely explanation is that the benefit is conferred by consuming fish types with the combination of relatively little mercury and high amounts of beneficial nutrients. This explanation is supported by results from multivariable models in the present analysis, in which adjustment for mercury strengthened the observed positive association of fish intake and cognition. Similarly, in the stratified analysis, we observed the highest cognitive scores among offspring of mothers with higher fish intake but lower mercury levels.

The fish questions in the Project Viva food frequency questionnaire were designed to estimate intake of fatty acids, not mercury. Because this questionnaire does not assess intake of individual fish types, but rather groups of fish, we cannot report associations for specific types of fish. Mercury levels vary among different fish species. In general, white meat fish such as cod and haddock tend to have lower mercury levels but also lower levels of long-chain n-3 fatty acids, whereas dark meat fish, such as swordfish, mackerel, and other large long-lived predatory fish, tend to contain both more mercury and more n-3 fatty acids. Because mercury and n-3 fatty acids often travel together, it may be difficult to isolate the opposing influences of the two on child cognition. Small fatty fish such as sardines and canned light tuna (vs. albacore tuna) may contain relatively more fatty acids with less mercury. Future studies incorporating more detailed dietary information may help advise women about specific fish species that are better or worse for their children’s cognition.

Resolving this issue remains important because women may indiscriminately reduce fish consumption in response to concerns about mercury exposure, perhaps substituting fish with other, less healthful foods. In a previous study, we demonstrated that a different subset of pregnant women enrolled earlier in our cohort reduced consumption not only of dark meat fish, which are likely to have higher mercury levels, but also of canned tuna and white meat fish, which tend to have lower mercury levels, after dissemination of a 2001 U.S. federal mercury advisory (Oken et al. 2003a). A recently updated federal advisory reiterated health warnings while encouraging women to consume up to two seafood meals per week (U.S. Department of Health and Human Services 2004), but it is unclear to what extent women understand the details of the health message or simply hear that seafood contains mercury and therefore is harmful.

In Project Viva, we assessed infant cognition using the VRM protocol. This test of cognitive function has many advantages for studies of prenatal exposures: It can be performed early in infancy and assesses cognition isolated from motor function. VRM tests have predicted IQ in childhood and early adolescence as strongly as other standardized tests of infant development (e.g., the Bayley Scale of Infant Development) (Rose and Feldman 1995; Rose et al. 1992), with correlations with intelligence in later childhood ranging from 0.44 to 0.66 (Laucht et al. 1994). However, similar to all tests of infant cognition, the VRM is most strongly correlated with later IQ when mental development is impaired, whereas the relationship is less strong when cognition is within the normal range. The VRM, which uses visual preference rather than motor skills to assess cognitive ability, might be particularly sensitive to the benefits of fish consumption: The marine fatty acid docosahexaenoic acid is an essential component of the retina and promotes infant vision (SanGiovanni et al. 2000). VRM testing has been used to demonstrate associations of infant cognition with infant intake of marine n-3 fatty acids (O’Connor et al. 2001; Uauy et al. 2001) as well as prenatal exposure to environmental pollutants such as lead and polychlorinated biphenyls (Darvill et al. 2000; Emory et al. 2003). Inverse associations of methylmercury exposure and novelty preference scores have been seen among non-human primates (Gunderson et al. 1986, 1988); however, other studies among human infants, most notably in the Seychelles Islands cohort, have not demonstrated an association of prenatal mercury exposure with VRM results (Darvill et al. 2000; Myers et al. 1995). Future cohort follow-up will help determine whether fish and mercury are associated with intelligence later in childhood.

Results should be generalized with some caution because our study population contained a high proportion of educated, white, and well-off mothers, all of whom lived in one area of the United States. Even within this sample, however, the main exposures and outcome were similar to those of other populations. For example, levels of hair mercury were similar to those in women of childbearing age in the most recent National Health and Nutrition Examination Survey (90th percentile, 1.4 ppm), which is nationally representative of the U.S. population (Centers for Disease Control and Prevention 2001). The mean novelty preference score of 59.8 in our population was similar to that observed among children enrolled in the Seychelles Child Development Study (60.3) (Myers et al. 1995) and among U.S. children living in New York State (59.8) (Darvill et al. 2000).

As with all observational studies, it is possible that we did not completely adjust for potentially confounding factors. For example, we did not collect information on parental IQ or home environmental stimulation, because Project Viva includes many outcomes of interest in addition to cognition, and we wished to reduce participant burden. Both of these factors might be associated with fish intake and have been associated with child cognition in prior studies, although usually only beginning in the later half of the second year of life. We also did not measure exposure to persistent organic pollutants such as polychlorinated biphenyls, although we would expect that accounting for the adverse effect of these toxicants would strengthen the observed benefit of seafood. We assessed mercury exposure using total maternal hair mercury, which is a recommended biomarker for estimating the methylmercury dose received by the offspring’s brain (National Research Council 2000) and strongly associated with both maternal blood and cord blood organic mercury (Morrissette et al. 2004). More than 80% of the mercury in hair is in the methyl form; in addition, organic mercury can be partly transformed to inorganic mercury, so the concentration of total mercury more accurately represents the mercury entering the hair follicle from the blood stream (Davidson et al. 1998; Myers et al. 2003). Some mercury may have derived from the inorganic mercury in dental amalgams. If this were true, however, the beneficial effect of fish on cognition then would be even stronger than what we observed. Our sample was too small to address whether there are subgroups within the population that are more susceptible to mercury exposure because of genetic, nutritional, or social factors that may sensitize the developing brain to neurotoxicants.

Results from the present study among a U.S. cohort with moderate fish intake suggest that maternal fish consumption during pregnancy may benefit offspring cognition in infancy but that exposure to higher levels of mercury has adverse effects on child cognition. These findings, based on a relatively small group of women, merit further investigation and verification in other populations consuming moderate amounts of seafood. Meanwhile, we recommend that women continue to consume fish during pregnancy but seek out varieties with lower levels of mercury.

## Figures and Tables

**Table 1:** Participant characteristics and their unadjusted associations with cognition in 6-month-old infants, assessed by VRM testing: results from 135 mother–infant pairs enrolled in Project Viva.

Maternal characteristics	Subjects (%)	VRM score (95% CI)	*p*-Value
Age (years)			0.80
< 30	16	60 (51 to 68)	
30–34	53	61 (57 to 65)	
≥35	31	59 (52 to 67)	
Race/ethnicity			0.81
White	82	60 (56 to 63)	
Nonwhite	18	61 (54 to 67)	
Marital status			0.79
Married or cohabitating	92	60 (57 to 63)	
Divorced or single	8	61 (51 to 70)	
Education level			0.25
College or graduate degree	80	59 (56 to 62)	
< College graduate	20	63 (58 to 68)	
Sex			0.56
Female	51	59 (55 to 62)	
Male	49	61 (56 to 66)	
Gestation length (weeks)			0.73
< 37	4	65 (50 to 79)	
37–38	22	58 (51 to 65)	
39–40	54	61 (57 to 65)	
≥41	19	58 (50 to 65)	
Birth weight for gestational age			0.76
Small (< 10th percentile)	2	53 (28 to 78)	
Appropriate	85	60 (57 to 63)	
Large (> 90th percentile)	13	59 (49 to 69)	
Breast-feeding duration (months)			0.23
< 2	19	54 (47 to 62)	
2–4	23	60 (53 to 67)	
≥5	58	61 (57 to 66)	
Infant age at testing (months)			0.37
< 7	80	60 (57 to 64)	
≥7	20	57 (51 to 64)	

**Table 2:** Associations of maternal second-trimester fish consumption and maternal hair mercury at delivery with infant cognition at 6 months (VRM score): results from six linear regression models among 135 mother–infant pairs in Project Viva.

	Change in VRM score [% novelty preference (95% CI)]	
Model	Effect per weekly fish serving	Effect per ppm maternal hair mercury
Fish only	2.5 (–0.01 to 5.0)	—
Fish and participant characteristics^a^	2.8 (0.2 to 5.4)	—
Mercury only	—	–4.6 (–10.3 to 1.1)
Mercury and participant characteristics^a^	—	–4.0 (–10.0 to 2.0)
Fish and mercury	3.9 (1.2 to 6.5)	–8.1 (–14.1 to –2.0)
Fish, mercury, and participant characteristics^a^	4.0 (1.3 to 6.7)	–7.5 (–13.7 to –1.2)

a Participant characteristics adjusted for include maternal age (continuous), race/ethnicity (white vs. nonwhite), education (college graduate vs. not), marital status (married or cohabiting vs. not), and infant sex, gestational age at birth (continuous), birth weight for gestational age (continuous), breast-feeding duration (continuous), and age at cognitive testing (continuous).

**Table 3:** Mean cognitive (VRM) scores (% novelty preference) among offspring of mothers with high or low second-trimester fish intake and high or low hair mercury levels at delivery.

	Hair mercury	
Weekly fish intake	≤1.2 ppm	> 1.2 ppm
> 2 servings	72 (n = 7)	55 (n = 2)
≤2 servings	60 (n = 114)	53 (n = 12)
